# Adapted Portuguese folk dance intervention for subacute rehabilitation post-stroke: study protocol

**DOI:** 10.3389/fpubh.2023.1200093

**Published:** 2023-08-17

**Authors:** Júlio Belo Fernandes, Josefa Domingos, Carlos Família, Júlio Veríssimo, Patrícia Castanheira, Carla Menezes, Célia Vicente, Claúdia Santos, Elisabete Marvão, Joana Coelho, Joana Mestre, Joana Teodoro, Júlia Saraiva, Marlene Cavaco, Natacha Sousa, Catarina Godinho

**Affiliations:** ^1^Egas Moniz Center for Interdisciplinary Research (CiiEM), Egas Moniz School of Health & Science, Almada, Portugal; ^2^Molecular Pathology and Forensic Biochemistry Laboratory (MPFBL), Almada, Portugal; ^3^UCCI São Roque, Santa Casa da Misericórdia de Lisboa, Lisbon, Portugal; ^4^Department of Nursing, Hospital Garcia de Orta EPE (HGO), Almada, Portugal; ^5^ARS LVT, ACES Lisboa Norte, UCC Integrar na Saúde—ECCI Benfica, Lisbon, Portugal

**Keywords:** stroke, sub-acute care, rehabilitation, dance therapy, exercise, aging, public health

## Abstract

Dance can be an innovative, well-accepted, and effective therapy for stroke survivors. The present protocol aims to assess the feasibility of adapted Portuguese folk dance as a post stroke rehabilitative activity. We will use a mixed-methods pilot study convenience sampling to recruit 16 stroke survivors with mild–moderate lower limb paresis from a rehabilitation center in Lisbon and Tagus Valley. In addition to usual care, participants will attend 3 days per week 1-h dance exercise for 8 weeks. The dance style used for this intervention will be an adaptation of the Portuguese folk dance. Assessment will be conducted before and immediately after the program intervention. Acceptability will be assessed by four key domains (enrollment, retention, satisfaction, and recommendation to others). Safety will be assessed based on the number and type of adverse events. Feasibility will be assessed based on balance performance and functional mobility. Quantitative data will be analyzed through descriptive statistics for sample characterization, followed by inferential statistics to evaluate differences in the balance recovery and functional mobility scores between the initial and final assessment. Qualitative data will be analyzed using an inductive process of content analysis. The Portuguese folk dance program has the potential to improve balance outcomes and functional mobility. Our results will help validate Portuguese folk dance as a tool for rehabilitation settings for stroke survivors. The potential of our program to enhance balance outcomes and functional mobility among stroke survivors bears implications for aging and public health initiatives. Positive results from this study could pave the way for integrating dance-based rehabilitative activities into standard stroke rehabilitation protocols, catering to older stroke survivors’ specific needs and preferences.

## 1. Introduction

Worldwide, stroke is the second leading cause of death and one of the leading causes of permanent disability (1, 2). As a result of the aging population and an increased survival rate, the absolute number of stroke survivors is rising (3). Many stroke survivors manifest some functional change (4). The most frequent impairments induced by stroke are centered on cognitive and motor disturbances, namely balance, coordination, proprioception, muscle tone, muscle strength, and gait (4, 5).

The 2016 American Stroke Association (ASA) and American Heart Association (AHA) guidelines recognize that stroke is a chronic condition and that many individuals require ongoing care to manage their needs. Therefore, they recommend expanding formal rehabilitation from the typical paradigm of 3–6 months (6). As a result of the rehabilitation process, many stroke survivors can regain functional capacity. Participation in rehabilitation programs that focus on balance and muscle-strengthening training is a factor that directly impacts the person’s recovery (7–9). However, limitations such as motor, visual, verbal, or informational memory loss, and impaired executive and social functions impact the patient’s ability to memorize the training steps and therefore hampers the effectiveness of rehabilitation programs (10).

In the last decade, numerous innovative rehabilitation interventions have emerged to address the needs of stroke survivors. Dance is increasingly used intervention to rehabilitate stroke survivors (11–15) Several investigations have shown that dance can be an effective treatment modality to address various impairments, with a relevant and sustained effect on psycho-emotional perspectives that can strongly enhance its use as rehabilitative activity (16–19).

Dance is a multidimensional intervention that exercises physical and motor skills and engages various cognitive functions such as perception, emotion, and memory (20). There is also evidence that dance can generate additional benefits in neurorehabilitation by inducing neuroplasticity (20).

For stroke survivors, dance can represent a therapy that enables enjoyable social activity and therapeutic exercise delivered at the appropriate intensity to improve balance, muscular strength, and mobility (12–15). In addition, dance can potentially improve stroke survivors’ self-esteem (21) and reduce the levels of psychological distress (22).

In Portugal, musical culture is part of the population’s roots. Portuguese folk dance is a group dance originating from the rural areas of Portugal. It is a movement art combining Portuguese folk songs with traditional folk dance. There are various kinds of dances stemming from different regions of Portugal. Nonetheless, although each dance employs a different technique, there are common traits in the various dances. All dances include circular movements and elaborate footwork, whether they consist of two or three steps, and dance in small circles or long lines (23, 24).

Considering the combination of complex and high-impact movements seen in Tango (15, 16) and Bolero (12) can improve the coordination and balance of patients with stroke, we hypothesize comparable outcomes with Portuguese folk dance.

Since folk dances are deeply rooted in the cultural heritage of a community, we expect that by incorporating a culturally specific intervention, stroke survivors can connect with their cultural identity, fostering a sense of belonging and familiarity. This can enhance motivation and engagement in the rehabilitation process. In addition, cultural activities have the potential to evoke positive emotions, such as joy, pride, and nostalgia (25, 26).

Therefore, engaging in familiar cultural practices, like folk dance, has the potential to reduce feelings of depression, anxiety, and social isolation (25). This positive emotional state can contribute to their overall well-being, motivation, and recovery.

This is the first know study that aims to find scientific evidence connecting Portuguese folk dance to balance and functional mobility improvement in stroke survivors. The implications of this study are manifold, particularly in the context of aging and public health. Aging populations increasingly face the challenges of stroke-related impairments, such as reduced balance and mobility, significantly impacting their quality of life. This study’s focus on stroke survivors addresses a crucial aspect of public health, as stroke incidence tends to rise with age, and effective rehabilitative interventions are essential for enhancing the well-being of this demographic. Therefore, our primary aim will be to assess the acceptability and feasibility of adapted Portuguese folk dance as a post-stroke rehabilitative activity.

## 2. Materials and methods

### 2.1. Study design

Program acceptability and feasibility will be evaluated with mixed methods using quantitative and qualitative assessments (27). To ensure quality in the research report, we followed the Good Reporting of A Mixed Methods Study (GRAMMS) checklist (28).

### 2.2. Study setting

The study will be conducted in the Portuguese National Network of Integrated Continued Care rehabilitation center. The rehabilitation center provides care for stroke survivors who have recovery potential but cannot return home due to stroke-related impairments after receiving acute care in hospital settings.

### 2.3. Sampling and recruitment

The sampling method selection will be non-probabilistic by convenience. All stroke survivors that comply with the study criteria will be invited to join the program.

We aim to recruit 16 participants taking into account a dropout rate of 25% (29).

Inclusion criteria:

1. 18 years old or above.
2. Clinically diagnosed with ischemic or hemorrhagic stroke.
3. Mild–moderate lower limb paresis with a modified functional ambulation classification (MFAC) of IV (assisted walker) or above.
4. A Montreal Cognitive Assessment (MoCA) score > 20.
5. Able to follow three-step directions.
6. Able to tolerate a minimum of 45 min of exercise.
7. Able to communicate with the investigator, to understand and comply with the study procedures.
8. Show a preference for Portuguese folk dance Vira.
9. Willing and able to provide written informed consent to participate and understand the right to withdraw their consent at any time without prejudice to future medical care.

Exclusion criteria:

1. Pre-existing neurological, cardiovascular orthopedic, or rheumatic disorder that could interfere with the execution of the proposed protocol;
2. Severe hearing and visual impairment;
3. Poor tolerance to a group setting or significant behavioral problems that would hinder the person from participating.

Two nurses from the rehabilitation center will be responsible for recruitment. In the first stage of recruitment, based on the data in the patient’s clinical file (diagnosis, stage of stroke, presence of hemiparesis, and clinical data), recruiters will identify stroke survivors as potentially eligible participants. In the second stage, a research team member will provide oral and written information regarding the study and recommend to each patient to take at least 24 h to reflect and discuss participation with their relatives/informal caregiver before deciding study to participate.

### 2.4. Intervention

The intervention consists of an 8-week program (1-h group training, three times per week). This intervention will supplement the usual care, which consists of daily 45 min sessions of occupational and physical therapy.

The dance style used for this intervention will be an adaptation of the Portuguese folk dance Vira, performed in every region of Portugal. The translation of the name is “twist, turn.” It has a three-step rhythm and is similar to a waltz. First, participants dance in pairs, front-to-front, holding arms in the air without holding hands, forming a circle that moves counterclockwise. At a certain point, half of the participants move to the center, where they tap their right foot and return to their respective places. Then, the circle starts to rotate again, alternating the participants who go to the center (23, 24).

Vira was selected because it combines whole-body movements requiring balance, flexibility, endurance, upper extremity function, perceptual-cognitive skills, and memory. Using Vira’s short routine will allow participants to repeat the dance steps requiring mobilization of attention and memory. In addition, the steps are simple, easy to learn and promote the transfer of weight from one side to the other.

Participants will be taught how to execute movements safely, focusing on maintaining proper alignment, balance, and coordination. Movements will be introduced gradually, allowing participants to build strength, flexibility, and control over time. Due to safety concerns, the dance program will incorporate modifications and adaptations to accommodate individual needs and limitations. Participants will not dance in pairs in this protocol. The instructor will pair participants with poor standing balance with the assistants. The assistants will support the patient by holding hands to increase stability. In addition, participants with poor standing balance will be provided with a gait belt to allow assistants to help them if necessary. We will modify the training for participants with severely reduced balance by dancing in a wheelchair. The instructor will encourage these participants to move their arms and legs, move the wheelchair forward and backward, and perform flexion/extension or lateral flexion of the trunk. The program will take place in a spacious, well-maintained environment with shock-absorbent and non-slip flooring.

Sessions will be led by a nurse specialized in rehabilitation expert in motor and cognitive exercise programs, and a background as a Portuguese folk dance instructor. We will consider eight stroke survivors as the optimum number of participants per session. Assistance to the instructor will be provided by three nurses that are receiving their master’s training in rehabilitation nursing, using a ratio of two participants for one therapist. In addition, the students will receive training to modify the exercises for each participant when needed and ensure their safety.

To provide a suitable level of challenge, the instructor will progressively increase the difficulty of the workouts accordingly to the participants’ willingness and improved condition. During sessions, the instructor will ask participants about their perceived effort through the Borg Effort Perception Scale (30). All professionals supervising the program application will provide the training’s adaptation, specificity, progression, variation, and safety. The structure of the training sessions is described in detail in Table 1.

**Table 1:** Structure of the training sessions.

Phase 1: Arrival (10 min)	Social interaction (e.g., greeting friends). A brief discussion on safety issues and how to participate (first-timers). Assess new health and issues linked to the sessions (second-timers).
Phase 2: Warm-up (10 min)	Group warm-up using whole-body amplitude movements incorporating neck, shoulders, hands, trunk, hips and knees movements, and slow passive stretching of the most affected upper extremity using the least affected upper extremity. Essential dance step (e.g., step touch) to increase heart rate. Walking in place or in chair, in a rhythmic routine using music based on group preferences. Voice warm up exercises with loud ahh, glides, and humming exercises.
Phase 3: Technical exercises (Two exercises per session; 10 min)	Trunk rotation. Step-touch side-ways. Moving the hips (side-ways). Weight shifting. Step-touch forward-backward. Basic step of the meringue. Sliding to the side. Twist.
Phase 4: Directed improvisation (10 min)	Participants will move freely according to some directions (bilateral movements only, large and big movements, etc.). In turn, they must either guide and be followed or follow the sequence proposed by another participant.
Phase 5: Dance routine (10 min)	Instructor will perform the routine, acting as a model for the participants. Integration of the dance routine with music. During the first 2 weeks, the sessions will focus on balance, requiring participants to form a circle, hold their arms in the air and transfer weight from one leg to the other. These moves will be followed by a couple of steps forward to reach the center of the circle, tap one of their feet and return to the starting position. From the third to the fifth week, in addition to the initial steps, participants will rotate 360° before moving to the center of the circle. In the last stage, participants will dance in a circle that moves counterclockwise.
Phase 6: Relaxation (5 min)	Active slow amplitude movements with music, stretching, and breathing exercises.
Phase 7: Session Assessment (5 min)	Brief group discussion to gather participant feedback on each exercise to guide future sessions.

### 2.5. Data collection

- Participant characteristics: Participants’ sex, age, educational level, occupation, stroke history, comorbidities, MFAC and MoCA scores will be extracted from the participants’ clinical records.
- Researchers defined acceptability a priori by four key domains for developing the program and performing a subsequent randomized controlled trial. These key domains include enrollment (number of stroke survivors that participate in the program), retention (number of classes attended and percentage of enrolled participants who completed final program assessments), and satisfaction (assessed with a survey and semi-structured exit interviews).

Safety via adverse events (number of events involving injury).

The study coordinator, who will not be involved in performing the intervention, will administer a customized post-intervention survey to assess participants’ acceptability of the intervention. The questionnaire will consist of Likert scale questions to evaluate the participants’ satisfaction. The interviews aim to gain insight into participants’ perspectives on the intervention’s acceptability and usefulness and identify exercise preferences, barriers, and facilitators that may influence the stroke survivor’s participation in the program.

The following outcome measures will be collected in the week before (T0) and the week after completing the training program (T1) by the following instruments:

- Balance recovery will be measured using the Mini-Balance Evaluation Systems Test (mini-BESTest), a 14-item performance-based clinical scale subdivided into four subscales that measure different domains of balance, including anticipatory postural control, compensatory postural control, sensory orientation, and dynamic gait. Each item is scored from 0 to 2. “0” indicates the lowest level of function, and “2” is the highest level of function (31). Previous studies have shown that the Mini-Balance Evaluation Systems Test is reliable and valid for evaluating balance in stroke survivors (32).
- Functional mobility will be measured using the Timed Up and Go (TUG) Test. The Timed Up and Go Test is a reliable instrument to assess the subject’s mobility and functional balance as it requires the ability to transfer, walk, and change direction (33, 34). Participants will be seated in a chair with their backs supported. At the evaluator’s indication, participants must stand and walk 3 m in a straight line, turn back, return to the chair, and sit down as in the starting position. The test score is according to the participants’ time to complete the task. A task completed within 10 s indicates normal mobility; 11–20 s are within normal limits for frail or disabled adults with partial independence, and greater than 20 s is expected for people with an important deficit in mobility (33).

### 2.6. Data analysis

#### 2.6.1. Quantitative evaluation

We will perform descriptive and inferential statistics of data collected at T0 and T1. Descriptive statistics, encompassing measures such as count, mean, standard deviation, minimum, first quartile, median, third quartile, maximum, interquartile range, and range, will be utilized to characterize the sample. The assessment of differences in balance recovery and functional mobility scores between the initial and final evaluations will be undertaken by means of inferential statistics. In particular, the parametric two-tailed paired samples *t*-test will be utilized if the normality assumptions are satisfied. In the event of deviations from normality, the non-parametric Wilcoxon signed-rank test will be employed. The statistical analysis will be performed using the R statistical computing software.

#### 2.6.2. Qualitative evaluation

Semi-structured interviews will be conducted by an experienced researcher, a skilled interviewer (35–37) with a Ph.D. in Nursing Sciences who has no prior relationship with the participants. Researchers will transcribe verbatim the audio-recorded interviews into a Word file. The data analysis will be conducted by two researchers independently. Textual data will be exported to the QDA Miner Lite database and analyzed using an inductive process of content analysis as described by Braun, Clarke, Hayfield, and Terry (38). This process will allow the identification of common themes, ideas, and patterns of meaning through pre-analysis, encoding, categorization, and interpretation of the data.

### 2.7. Ethics and dissemination

Researchers will conduct this research following the Helsinki Declaration (as revised in 2013) and seek approval from the rehabilitation center Ethics Committee. Participants will be asked to sign an informed consent prior to any procedures. The informed consent form will include comprehensive information regarding the study aims, procedures, voluntariness, and possible risks of participation. Researchers’ will emphasized that the participant has the authority to withdraw their consent to participate at any time without consequence or loss of benefits to which the participant is otherwise entitled. All information will be kept strictly confidential. All information will be destroyed 5 years after completion of the research project.

## 3. Discussion

This pilot study will assess the feasibility of adapted Portuguese folk dance as a post stroke physical activity. We expect that an adapted Portuguese folk dance training program will challenge the balance system and be effective for balance outcomes and functional mobility, increasing the Mini-BESTest score and the Timed Up and Go Test.

Data from previous studies suggest that dance can be a rehabilitation intervention that generates health benefits (12, 13, 15). However, despite the positive health outcomes of dance therapy for older adults (39, 40) and people with Parkinson’s disease (41–43), it is essential to highlight that few studies using dance therapy, including stroke survivors, were randomized controlled trials. It is also noteworthy that from the few studies that have examined dance in stroke survivors, a majority have been conducted in the chronic phase.

Previous studies have identified several barriers to stroke survivors’ participation in rehabilitation programs (44, 45). Therefore, developing new therapeutic approaches that are technically feasible, economically valuable, and culturally, ethically, and socially accepted among stroke survivors is crucial.

Incorporating Portuguese folk dance in rehabilitation training is innovative. In addition, it has the potential to achieve better patient health outcomes and motivate stroke patients to adhere to the rehabilitation program.

Dance seems to have the potential to be an intervention adapted to the multidimensional impairments of stroke survivors (e.g., cognitive, motor, balance, and social impairments) (46). Research is essential to determine the effects of dance intervention on stroke survivors’ balance and gait. As such, the first step is establishing a dance program’s feasibility for this population (27).

Since this pilot study aims to determine the program’s acceptability and feasibility, our results will not allow us to determine that the participants’ health outcomes are due to the dance intervention. However, this study precedes the development of a randomized controlled trial that may prove the efficacy of adapted Portuguese folk dance as a therapeutic intervention for patients with stroke. If proven effective, the results should benefit stroke survivors by safely improving their balance and gait patterns. Additional benefits over clinical disease management, cognition, physical capacity, depression, and fear of falling are also expected. This will ultimately promote Portuguese folk dance as a rehabilitation intervention for stroke survivors, opening the door to testing its effectiveness, and acceptability in other populations in worldwide healthcare systems.

Although dance can be enjoyable, it can also be intimidating for people who have not previously participated in dance classes (47). However, incorporating Portuguese folk dance, part of the population’s cultural roots, in rehabilitation training can increase Portuguese stroke survivors’ adherence. In addition, this intervention has the potential to promote social interaction, as it will be performed in a group format. Therefore, we expect the participants to develop a sense of camaraderie (13, 14, 48) and accomplishment (14), contributing to the intervention’s satisfaction (13, 14). Given all these aspects, we believe incorporating Portuguese folk dance in rehabilitation training should be an especially well-adapted and attractive way of optimizing patient recovery after stroke.

As in previous studies that use dance intervention for stroke survivors in rehabilitation hospital settings, we expect the level of risks associated with the intervention to be low (14). However, the potential risks of a dance intervention should be anticipated and addressed to guarantee participants’ safety. Therefore, the instructor can adapt the exercise program and choreography to keep each participant’s skills and abilities challenging and engaging. In addition, as a safeguard, we will consider eight stroke survivors as the ideal number of participants for each session, depending on the stroke survivors’ dependence, thus achieving a ratio of two participants for one therapist.

This study is not without limitations. First, the protocol will not include a control group, which will prevent comparing the effect of the dance intervention to additional treatment. Second, since stroke survivors are recruited in the subacute phase, some participants may suffer disease complications that stop them from attending the program.

Third, when assessing the program feasibility, we acknowledge the possibility of social desirability bias, as participants might report perceptions and feelings that might diverge from their emotions. Therefore, to minimize social desirability during the qualitative assessment, we will implement practices recommended by Bergen and Labonté (49). The interviews will be performed in a private location and not within earshot of others. The interviewer will use various approaches to establish rapport with participants, including humor, self-disclosure, and/or making displays of respect. We will clearly explain details about the study to participants, including how the data will be used and confidentiality and anonymity procedures. Suppose the interviewer suspects a response reflects social desirability tendencies. In that case, he will maintain a nonconfrontational and respectful posture and try to elicit a more accurate response by providing context when asking questions, acknowledging that people have diverse experiences, posing indirect questions, and requesting that participants provide a story or example to illustrate their response.

## 4. Conclusion

There is a growing interest in using dance as a therapeutic intervention for stroke survivors. This study aims to establish the acceptability and feasibility of adapted Portuguese folk dance as a post stroke rehabilitative activity. It is possible to identify that the current literature has no studies on the use of Portuguese folk dance in rehabilitation settings. Therefore, this will be the first study to assess this adapted dance program’s feasibility and preliminary effects. The results of this research will contribute to new scientific knowledge, supporting the use of Portuguese folk dance in rehabilitation settings as an effective tool for treating stroke survivors. Ultimately, validating Portuguese folk dance as a tool for rehabilitation settings offers a culturally meaningful and engaging approach to post-stroke care. Such findings can potentially improve stroke survivors’ well-being and foster cross-cultural exchange in stroke rehabilitation practices, thus benefiting aging populations worldwide.

## Author contributions

JF, JD, CF, JV, PC, CM, NS, and CG: conceptualization. JF, JD, CF, JV, PC, CM, CV, CS, EM, JC, JM, JT, JS, MC, NS, and CG: methodology, writing—original draft preparation, and writing—review and editing. JF and CG: supervision. JF: project administration. All authors contributed to the article and approved the submitted version.

## Conflict of interest

The authors declare that the research was conducted in the absence of any commercial or financial relationships that could be construed as a potential conflict of interest.

## Publisher’s note

All claims expressed in this article are solely those of the authors and do not necessarily represent those of their affiliated organizations, or those of the publisher, the editors and the reviewers. Any product that may be evaluated in this article, or claim that may be made by its manufacturer, is not guaranteed or endorsed by the publisher.
